# Biomarkers in Heart Failure

**DOI:** 10.5935/abc.20190167

**Published:** 2019-08

**Authors:** Pedro Pimenta de Mello Spineti

**Affiliations:** Hospital Universitário Pedro Ernesto, Rio de Janeiro, RJ - Brazil; Hospital Unimed-Rio, Rio de Janeiro, RJ - Brazil

**Keywords:** Biomarkers, Lipoproteins, Heart Failure, Hypertension, Diabetes Mellitus

The World Health Organization defines biomarker as any substance, structure, or process that can be measured in the body or its products and influences or predicts the incidence or outcome of a disease.^1^Biomarkers can serve multiple purposes: diagnostic, disease staging, prognostic and prediction and monitoring of responses to an intervention.^1^

A useful biomarker should allow repeated and accurate measurements with a rapid turnaround time at a reasonable cost, should provide information that is not already available from careful clinical assessment and its performance should be superior to other available tests, and should assist decision making and enhance clinical care.^2^

Several biomarkers have been studied in the context of acute and chronic heart failure (HF). In 2016 the American Heart Association issued a statement on the Role of Biomarkers for the Prevention, Assessment, and Management of Heart Failure.^3^ After an extensive review, they stated that a number of biomarkers associated with HF are well recognized, and measuring their concentrations in circulation can be a convenient and noninvasive approach to provide important information about disease severity and help in the detection, diagnosis, prognosis, and management of HF. These include natriuretic peptides, soluble suppressor of tumorigenicity 2 (ST-2), highly sensitive troponin, galectin-3, mid regional pro adrenomedullin (MR-proADM), cystatin-C, interleukin-6 and procalcitonin. There is a need to further evaluate existing and novel markers for guiding therapy.

The 2018 Brazilian Guidelines on Chronic and Acute Heart Failure recommends the use of natriuretic peptides with diagnostic and prognostic purposes.^3^ According to these guidelines other biomarkers such as troponins T and I, galectin-3 and ST-2 may add prognostic information in HF patients.^4^

More recently Swedish investigators reported that elevated plasma levels of NT-proBNP, MR-proADM, copeptin, and cystatin C were associated with higher mortality after discharge in a cohort of 286 patients hospitalized for newly diagnosed or exacerbated HF.^5^ Nonetheless, NT-proBNP was the only biomarker to predict the risk of re-hospitalization due to cardiac causes.

Lipoprotein(a) (Lp(a)) is a biomarker associated with increased risk of atherosclerotic disease. In 2016 Kamstrup and Nordestgaard demonstrated a clear stepwise association of elevated Lp(a) levels with increased risk of HF in a study with more than 98,000 danish participants.^5^ In addition, they provided genetic evidence that this association was mediated at least partly via coronary heart disease (CAD) and aortic valve stenosis.

This issue of *Arquivos Brasileiros de Cardiologia* presents the paper of Jianlong et al.^6^ examining the prognostic value of Lp(a) in Chinese patients admitted for decompensated HF of ischemic origin. An Lp(a) greater than 20,6 mg/dL was associated with 3 fold increase in readmission for HF. Patients with higher Lp(a) levels also had higher NT-proBNP levels, higher NYHA class, lower left ventricle ejection fraction, more CAD. The results were adjusted for theses covariates with a slight decrease in the hazard ratio.

Lp(a) may be a prominent new biomarker in patients with HF of ischemic origin. More studies in different populations are needed to validate these results.
